# A reply to ‘Metabolic effects of sapropterin treatment in autism spectrum disorder: a preliminary study'

**DOI:** 10.1038/tp.2016.24

**Published:** 2016-04-26

**Authors:** K Fluegge

**Affiliations:** 1 Institute of Health and Environmental Research, Cleveland, OH, USA

Frye *et al.*^1^ published a study in *Translational Psychiatry* that indicated that treatment with sapropterin, which is a synthetic form of tetrahydrobiopterin (BH4), improved metabolic outcomes in patients with autism spectrum disorder (ASD). They explained that BH4 is a critical co-factor for the production of precursors of many monoamine neurotransmitters, including dopamine (DA)^2^ and norepinephrine (NE),^3^ and is vital in nitric oxide (NO) production. The author of this letter has published an ecological investigation that may shed light on the existence of BH4 in ASD and why supplementation appears to ameliorate behavioral and metabolic outcomes in ASD, as shown by Frye *et al.*^1^

The increase in NO levels that is often noted in ASD may have to do with the parasympathetic dominant state that arises from chronic gestational exposure to nitrous oxide (N_2_O) in the environment, most especially from agricultural practices but other sources as well, as discussed elsewhere.^4^ This cycle may start, given that N_2_O, at clinically relevant doses, inhibits the human (alpha 7) nicotinic acetylcholine receptor (alpha 7 nAChR)^5^ and results in elevated central levels of DA and NE, as discussed previously.^4^ Others have found this particular nicotinic ACh receptor subtype to be altered in ASD.^6, 7^ Low concentrations of monoamine uptake inhibitors (that is, elevated synaptic NE) enhanced cerebral vasodilation mediated by alpha 7 nAChR,^8^ suggesting that elevated central NE levels may overcome central N_2_O-mediated inhibition of this receptor. Alpha 7 nAChR also acts as an anti-inflammatory in the periphery, and activation of the receptor prevented H_2_O_2_-mediated cell damage,^9^ suggesting that early gestational inhibition of alpha 7 nAChR may contribute to a higher oxidative stress baseline in ASD subjects.

Therefore, if gestational exposure to N_2_O perturbs, among many targets,^4^ alpha 7 nAChR activity, an uncoupling from eNOS may also occur,^10^ facilitating the production of H_2_O_2_, which can enhance cerebral endothelial ‘agonist-induced vasodilation' induced by acetylcholine.^11, 12^ The cholinergic system may, therefore, have a key etiological role in a mouse model of ASD.^13^ This cascade is dependent upon increased superoxide dismutase and decreased catalase, which characterize oxidative stress profiles in patients with ASD.^14^ H_2_O_2_ has been shown to induce a long-lasting bradycardia in rats that was inhibited by catalase activity^15^ and stimulated BH4 synthesis in vascular endothelial cells,^16^ although the magnitude of alpha 7 nAChR impairment during gestational N_2_O exposure may impact the capacity of central stimulation in ASD patients.^17^ Nevertheless, higher H_2_O_2_ production (indicative of gestational N_2_O burden) may help to explain increased plasma levels of NO^18^ in ASD. Moreover, Wu *et al.*^19^ reported that GTS-21, an alpha 7 nAChR agonist, ‘inhibited the production of IFN-γ by PBMCs from patients with RA in a dose-dependent manner and reduced the levels of IFN-γ to levels similar to, or even below, those found in healthy volunteers', suggesting that inhibition of this particular nAChR subtype may contribute to not only elevated plasma NO in ASD but also increased inflammatory markers, like IFNγ, as has been shown.^18^ These studies support the claim by Frye *et al.*^1^ that ‘the increase in NO metabolism seen in some individuals with ASD is associated with greater morbidity and a less favorable prognosis'.

The author has previously discussed the other physiological roles of N_2_O, including the inhibition of dopamine 4 receptor (DR4) activity through impairment of methionine synthase.^4^ The elegant studies of Yuen and Yan^20^ suggest that DR4 activation exerts an activity-dependent control of calcium homeostasis that then has a bi-directional impact on glutamatergic signaling in pyramidal neurons of prefrontal cortex, potentially contributing to autonomic dysregulation. Furthermore, Koyanagi *et al.*^21^ reported that the antinociceptive effect of N_2_O was mediated in part by dopamine receptor 2, and activation of this receptor could be expected to promote parasympathetic tone.^22, 23^ Therefore, disruption of these many intricate control mechanisms, perhaps through chronic gestational environmental N_2_O exposure, may confer a parasympathetic dominance.

The BH4 dysregulation in ASD may be a manifestation of this parasympathetic dominance to accommodate a low-grade N_2_O (that is, κ-opioid) dependence developed *in utero*. Recent studies that intimate a sympathetic dominance in ASD^24, 25, 26^ may actually be revealing the paradigm of opiate withdrawal in ASD subjects,^27^ especially given the seasonality of agricultural N_2_O emissions.^28^ Given that 3CT is a known inhibitor of tyrosine hydroxylase,^29^ a rate-limiting enzyme involved in catecholamine synthesis, the significant decrease in 3CT after supplementation^1^ may indicate the role of BH4 in the restoration of myogenic and central catecholaminergic activity, much like naltrexone,^30, 31^ an opioid antagonist. These contributions may help to explain the amelioration of behavioral (that is, irritability, hyperactivity) and metabolic (that is, NO) outcomes characteristic of ASD patients.
